# C-Peptide and leptin system in dichorionic, small and appropriate for gestational age twins—possible link to metabolic programming?

**DOI:** 10.1038/s41387-020-00131-2

**Published:** 2020-08-10

**Authors:** Krzysztof C. Lewandowski, L. Biesiada, M. Grzesiak, A. Sakowicz

**Affiliations:** 1 Department of Endocrinology and Metabolic Diseases, Polish Mothers’ Memorial Hospital—Research Institute, Lodz, Poland; 2 Medical University of Lodz, Department of Endocrinology and Metabolic Diseases, Lodz, Poland; 3 Department of Obstetrics and Gynecology, Polish Mother’s Memorial Hospital—Research Institute, Lodz, Poland; 4 Department of Perinatology, Obstetrics and Gynecology, Polish Mother’s Memorial Hospital—Research Institute, Lodz, Poland; 5 Medical University of Lodz, Department of Medical Biotechnology, Lodz, Poland

**Keywords:** Pre-diabetes, Endocrine system and metabolic diseases

## Abstract

Children born small for gestational age (SGA) are at increased risk of future glucose intolerance and type 2 diabetes, possibly after due intrauterine metabolic programming. Soluble leptin receptor (SLR) limits leptin access to signal-transducing membrane receptors. The present study examines whether SGA and appropriate for gestational age (AGA) twins differ with regard to their C-peptide, glucose and leptin systems. The markers C-peptide, glucose, fetal leptin, and SLR in cord blood were assessed in children from dichorionic twin pregnancies at delivery. In 32 cases, weight differed by >15% between twins: one demonstrated Intrauterine Growth Retardation (IUGR) (<10th percentile-SGA), while the other did not (AGA_I_). The other 67 pairs presented appropriate weight for gestational age (AGA_II_). Placental leptin and placental leptin receptor content were also assessed. Despite the same concentrations of glucose, the SGA twins maintained a higher level of C-peptide [44.48 pmol/l vs. 20.91 pmol/l, *p* < 0.05] than the AGA_I_ co-twins, higher HOMA index, calculated as [C-peptide] x [Glucose] (*p* = 0.045), in cord blood, and a higher level of SLR [SGA vs AGA_I_—mean: 28.63 ng/ml vs. 19.91 ng/ml, *p* < 0.01], without any differences in total leptin (*p* = 0.37). However, SGA placentas demonstrated higher leptin level [130.1 pg/100 g total protein *vs* 83.8 pg/100 g total protein, *p* = 0.03], without differences in placental leptin receptor (*p* = 0.66). SGA/IUGR twins demonstrate relative insulin resistance accompanied by decreased fetal and increased placental leptin signaling. We speculate that relative insulin resistance and changes in the leptin system might be the first evidence of processes promoting deleterious metabolic programming for post-natal life.

## Introduction

Since the late 1980’s, it has been known that children born small for gestational age (SGA) have an increased risk of cardiovascular disease in future life^1,2^, thus leading to the formulation of the “developmental origins of health and disease” (DOHaD) hypothesis^3^. According to DOHaD, an organism exposed to undernourishment in the uterus diverts the restricted nutrients to preserve the growth and function of vital organs, such as brain, at the expense of growth and organs, such as liver and pancreas. Though such intrauterine adaptation in conditions of inadequate nutrition is favorable for survival, this also has its consequence for the postnatal life through modulation of phenotype; i.e., the so-called “thrifty phenotype hypothesis”^4^. Intrauterine growth restriction (IUGR) is often detected near mid-pregnancy in women and persists until term. Both maternal and placental factors are believed to influence IUGR development, with the key maternal risk factors being hypertension, diabetes, metabolic and chronic diseases, smoking, low maternal weight during pregnancy and social economic status^5^. The risk of placental insufficiency is associated with local disturbances in the delivery of oxygen and nutrients into the developing fetus caused by abnormal (shallow) implantation of trophoblast cells into maternal decidua^5^.

In the 1990’s, it was noted that IUGR is commonly associated not only with an increased incidence in perinatal mortality but also with an elevated risk of chronic metabolic diseases (such as obesity and type 2 diabetes) later in life, potentially reflecting incorrect “metabolic programming”^6^. Though low birth weight in twins may be associated with an increased risk of type 2 diabetes, it is not presently clear whether alterations in glucose homeostasis are already visible at birth^7^.

In dichorionic pregnancies, both fetuses develop independently of each other, so the “stealing” problem characteristic of monochorionic pregnancies generally does not appear. The two thrive in the same maternal conditions, and so only the local environment related to placental development may influence the supply of nutrients and oxygen transfer. In such a context, dichorionic twin pregnancy constitutes a perfect model for the assessment of potential metabolic alterations contributing to metabolic programming for future life.

As C-peptide is secreted from the beta cells of pancreas in equimolar ratio with insulin, its level accurately reflects insulin secretion. In contrast to insulin, C-peptide is not extracted by the liver and other organs, and the half-life of it in blood is much longer than insulin (10–30 vs. four minutes). Therefore, C-peptide levels reflect endogenous insulin secretion more accurately than insulin concentrations^8^. Furthermore, the cord serum level of C-peptide is more commonly used as an index of fetal beta-cell function than insulin levels, because degradation of insulin might be increased in the presence of even slight hemolysis^9^.

The leptin system consists of free leptin, membrane leptin receptors and the soluble leptin receptor^10^. In this system, insulin infusion stimulates an increase in free leptin concentration^11^. Leptin expression has been described not only in maternal circulation but also in placenta and in umbilical cord blood^6,12^. The action of leptin depends not only on the availability of its receptor on the cell membrane, but also on blood content of the soluble leptin receptor. When higher concentrations of soluble receptor are observed in the blood, it has been found that less leptin is available for binding to the membrane form of its receptor. This leads to the down-regulation of leptin signaling because an increase of concentrations of soluble leptin receptor might impede leptin binding to signal-transducing membrane receptors^13^.

Depending on the stage of life, the leptin system can play various roles. In postnatal life, the leptin system directly influences the central nervous system to adjust the food intake to the energy expenditure. Moreover, leptin is implicated in the regulation of energy homeostasis, neuroendocrine metabolism and carbohydrate metabolism, including the regulation of glucose turnover and insulin responsiveness^14^. Placental leptin appears to inhibit apoptosis and promote the growth, proliferation and cell survival of trophoblastic cells by activating the JAK-STAT, MAPK and PI3K signaling pathways^15^. Moreover, it is believed that leptin secreted by the placenta acts as a modulator of maternal inflammatory and immune responses, thus preventing embryo rejection^16^. In turn, fetal leptin exerts a pleiotropic role: it is responsible for fetal skeletal development and maturation of the fetal immune system, stimulating myelopoiesis, erythropoiesis and lymphopoiesis^17,18^. Furthermore, it was observed that leptin may play a lipostatic role before birth. Leptin-infused sheep fetuses show lower number of unilocular and higher number of multilocular fat cells^19^.

Hence, we hypothesize that in human fetuses, down-regulation of leptin access to the cell, stemming from a reduction of the free leptin pool, either through lower leptin blood concentration or through elevated concentrations of soluble leptin receptor, its natural inhibitor, may protect the small for gestational age (SGA) fetus against unfavorable reduction of the number of unilocular fat calls.

Furthermore, a negative correlation has been reported between leptin level and cortisol in sheep fetuses^20^, despite the fact that postnatally glucocorticoids directly stimulate leptin release^11^ and glucocorticosteroids constitute one of the strongest promotors of insulin resistance. Therefore, we speculate that dysregulation of leptin homeostasis in SGA fetuses may help them maintain the appropriate level of glucose essential to nourish the vital organs for survival.

Therefore, the present study examines the glucose homeostasis (C-peptide and glucose) and the leptin system (i.e., fetal total leptin and soluble leptin receptor) in human fetuses from dichorionic twin pregnancies, where only one twin was affected with IUGR. In our study, both fetuses thrived independently of each other under the same maternal conditions and only the local environment influenced the development of IUGR. Therefore, we propose that disturbances in concentrations of the studied markers may indicate that in SGA fetuses^1^, a predisposition towards future glucose intolerance/diabetes mellitus has its origins in utero and^2^ the metabolism is also programmed towards the “thrifty phenotype” in utero.

## Subjects and methods

### Subjects

We recruited all patients with dichoronic twin pregnancy who consented for testing. The study included 32 dichorionic twin pairs, where one twin had evidence of IUGR (SGA twin), defined as weight difference between twins exceeding 15%, where the weight of the larger twin was adequate for gestational age (AGA_I_ twin). In addition, one twin was small for gestational age (<10th percentile-SGA) (IUGR), while the other was appropriate for gestational age (>10th percentile–AGA_I_). The second group included 67 dichorionic twin pairs, where both twins were adequate for gestational age, and the weight difference between twins was <15% (AGA_II_ twins).

The exclusion criteria for both groups comprised the presence of chromosomal abnormalities, placental vascular abnormalities and infarction. Premature rupture of membranes (PROM), pregnancy-induced hypertension, maternal hypothyroidism and gestational diabetes mellitus did not exclude the patients from the study. Both groups included similar proportions of women who had conceived by IVF, delivered twins by Caesarian section or vaginally, and both groups included similar proportions of fetuses with regard to sex. i.e., male–male, female–female, male–female. Dichorionic twin pregnancy was identified based on ultrasonography conducted in the first trimester of gestation.

Demographic characteristics of the investigated group is presented in Table 1.

**Table 1:** Demographic characteristics of the investigated groups. SGA & AGA group: weight difference between twins from dichorionic pregnancy >15%, smaller twin with evidence of IUGR. AGA_II_ group: weight difference between twins from dichorionic pregnancy <15%.

Parameter	SGA + AGA_I_ Group (*n* = 64)	AGA_II_ Group (*n* = 134)	*p*-Value
Fetal weight [g], *n* = 32 + 32	2216 ± 510	2357 ± 417	0.194*
Fetal weight [g] SGA_I_, *n* = 32	1944 ± 413		0.000003*
Fetal weight [g] AGA, *n* = 32	2479 ± 444		0.151*
1st minute Apgar score	8.28 ± 1.38	8.72 ± 1.12	0.016*
Week of delivery	35 ± 2.1	35 ± 1.9	0.422*
Male sex (%)	55%	52%	0.728**
Female sex (%)	45%	48%	

*Presented as mean ± SD. *p*-Value calculated by the means of Student *t*-test.

**p-Value calculated by the means of chi^2^ test.

All patients were recruited from the Polish Mothers’ Memorial Hospital - Research Institute from 2013 to 2018; all received standard clinical care. The Institution serves as the main secondary referral center for the Lodz region with a catchment area population of over two million. The presence of dichorionic twin pregnancy was confirmed by relevant antenatal ultrasound examination. Informed consent was obtained from all participants. All patients with dichorionic twin pregnancy who arrived for delivery in the Institution were asked to take part in the study, and data for all analyzed parameters was acquired from these patients (*n* = 99). Nine of these patients refused to consent to data collection and were excluded from the study. The study was approved by the Ethics Committee of the “Polish Mothers’” Memorial Hospital Research Institute - decision number 85/2013.

### Collection of blood and placental samples

Umbilical cord blood was taken from the vein of umbilical cord immediately after delivery. The samples were centrifuged at 3000 rpm for 10 min and serum was divided into 5–6 Eppendorf tubes. The serum samples were stored at −80 °C for further examination.

Placenta samples were taken immediately after delivery. Fragments of about 2 cm^3^ were trimmed under the place of trailers of umbilical cord. The samples were stored in RNALater (Ambion, USA) at −80 °C for further analyses. Whole placental protein fraction was isolated using PBS pH 7.2 mixed with Halt^TM^ Protease Inhibitor Cocktail (Thermo Scientific, USA). The concentration of protein was measured using Pierce™ BCA Protein Assay Kit (Thermo Scientific, USA) according the manufacturer’s protocol. The total protein fractions were stored at −80 °C for further examination.

### Study protocols

The Enzyme Linked-Immunosorbent Assay (ELISA) tests were conducted for quantitative determination of Leptin (Cloud Clone, USA; SEA084Hu), intraassay variation 2%, interassay variation 10.6%, soluble Leptin Receptor (BioVendor, Czech Republic; RD194002100), intraassay variation 1.9%, interassay variation 3.4% and C-peptide (Abebio, China; AE49173HU). We also measured ultrasensitive C-peptide (Creative Diagnostics, USA; DEIA-CL003U), intraassay variation 9.0%, interassay variation 8.1% in serum of umbilical cord blood. These coefficient variations are regarded as acceptable by FDA recommendations^21^.

Additional, the following tests were used for assessment of Leptin and its receptor in the whole cell lysates of placental samples: Leptin (Cloud Clone, USA; SEA084Hu) and Leptin Receptor (Cloud Clone, USA; SEA083Hu). Microtiter plates coated with antibody against the appropriate protein were incubated with the serum or whole placental lysates of patients. The time and temperature of incubation depended the type of analyzed protein: one hour at 37 °C for Leptin and Leptin Receptor (Cloud Clone) and C-peptide, one hour at 25 °C for soluble Leptin Receptor (BioVendor) and 2 h at 25 °C for ultrasenstive C-peptide test. The plates were washed by Wash Buffer after incubation. Following the wash, the plates were incubated with biotin conjugated antibody against the studied proteins. The plates were incubated according to the manufacturers’ protocols (time and temperature appropriate to protein type), and then were washed and incubated with Streptavidin-HRP Conjugate for 30 min. The plates were then incubated with Substrate Solution. The reactions were stopped by Stop Solution and absorbance for each plate was determined at 450 nm.

Glucose and C-peptide product levels were calculated as HOMA-IR equivalents, as originally described by Matthews et al.^22^.

### Statistical data analysis

Statistical analyses were performed using Statistica version 13.1 (Statsoft, Poland). The sample size of 32 pair of twins (64 patients) has been shown to have a statistically significant impact on the assessment of insulin resistance and endocrine disorders both for the twin and singleton gestation population studies^23,24^. The distribution of the analyzed data was checked by Shapiro–Wilk test. Normally-distributed data were further analyzed with the two-sided Student’s *t*-test, the equality of variances for a variable calculated for two groups was assessed by Levene’s test. The non-normally distributed data were analyzed using the two-sided Mann–Whitney *U*-test. Cases with missing values are not included in the analyses. The categorical variables were calculated by the chi-square test or with Yates’s correction. A *p*-value below 0.05 for the test results was considered as statistically significant.

### Results

**Fig. 1:**
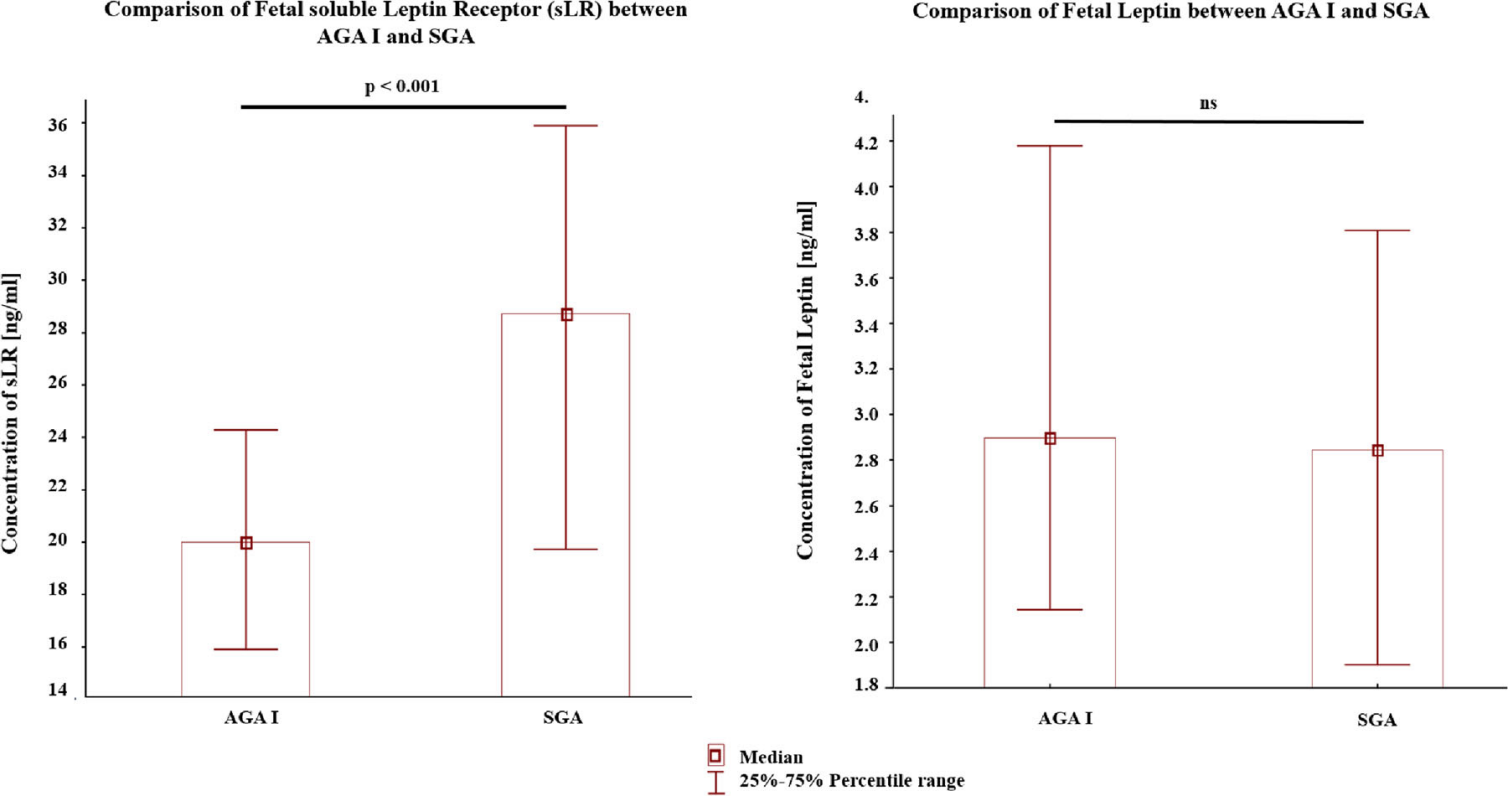
Comparison of concentrations of fetal soluble leptin receptor and fetal total leptin between twins with evidence of in intrauterine growth retardation (IUGR)—one small for gestational age (SGA) and the other appropriate for gestational age (AGA_I_) from a dichorionic pregnancy. Data were tested with the Mann–Whitney U-test; a *p*-value of < 0.05 indicates significant results; ns-not significant.

**Fig. 2:**
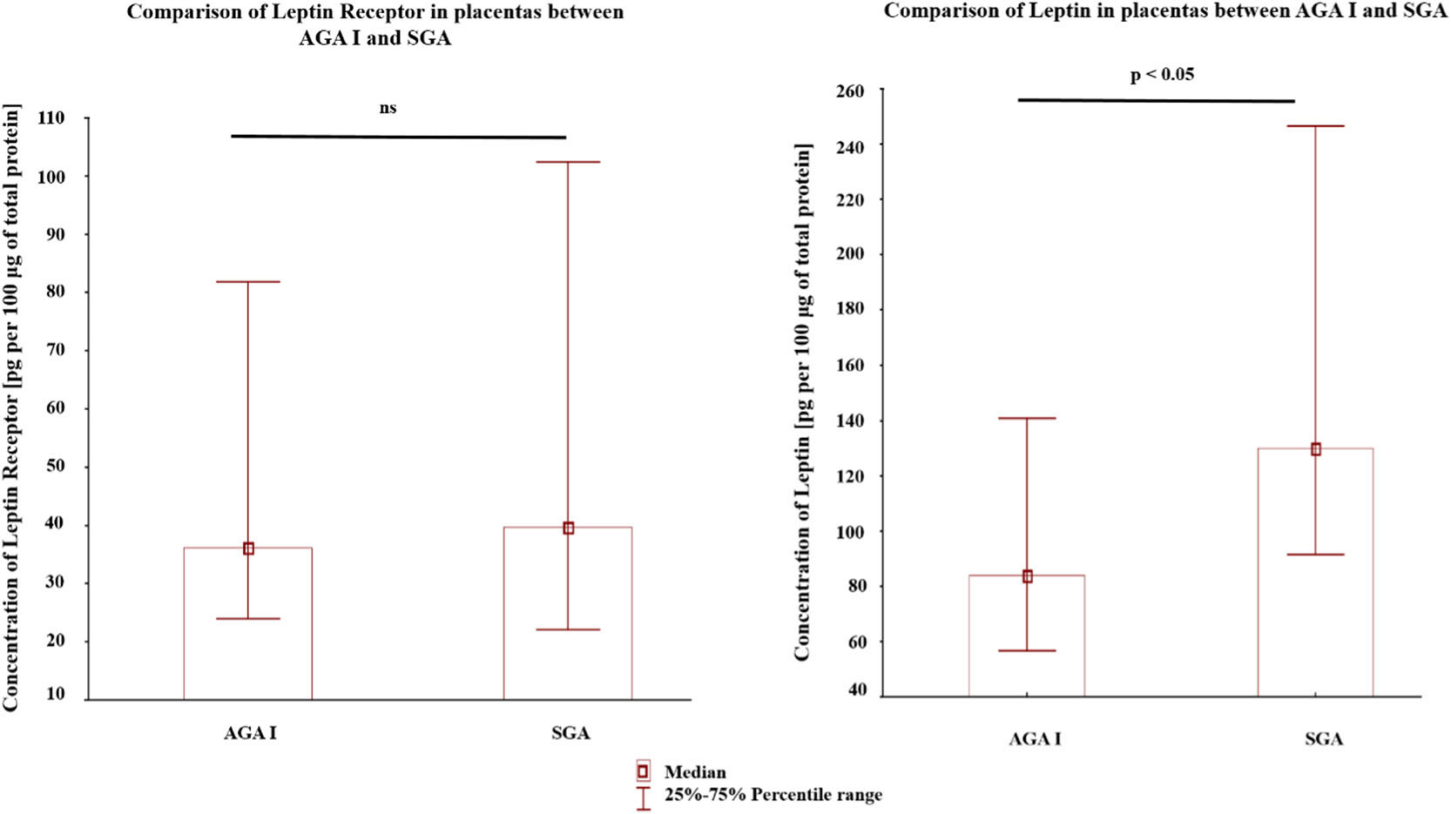
Comparison of content of placental total leptin receptor and placental leptin between twins with evidence of in intrauterine growth retardation (IUGR)—one small for gestational age (SGA) and the other appropriate for gestational age (AGA_I_) from a dichorionic pregnancy. The results were recalculated accordingly: nanograms [ng] of studied protein per 100 μg of total protein isolated from the placental sample. Data were tested with the Mann–Whitney *U*-test; a *p*-value of < 0.05 indicates significant results; ns-not significant.

**Fig. 3:**
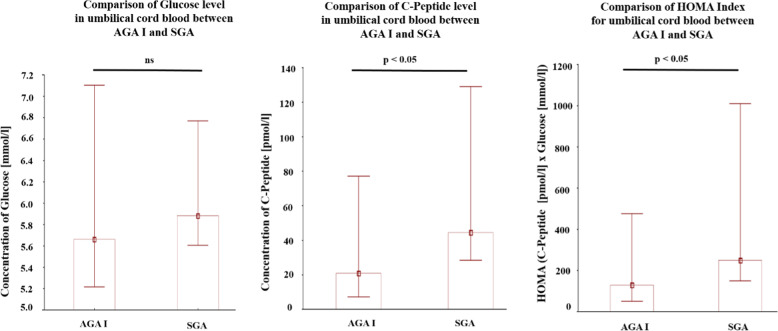
Comparison of concentrations of glucose, C-peptide and Glucose × C-peptide product (HOMA-IR equivalent) between twins with evidence of inintrauterine growth retardation (IUGR)—one small for gestational age (SGA) and the other appropriate for gestational age (AGA_I_). Data were tested with the Mann–Whitney *U*-test; a *p*-value of < 0.05 indicates significant results; ns-not significant.

Results of the investigated parameters from the SGA & AGA_I_ group (*n* = 32 each) are presented in Table 2 and Figs. 1–3.

**Table 2:** Analysis SGA vs AGA_I_ from the same pregnancy (*n* = 32).

Parameter	SGA Median (interquartile range)	AGA_I_ Median (interquartile range)	*p*-Value
Fetal soluble leptin receptor [ng/ml]	28.63 (19.64–35.82)	19.91 (15.83–24.21)	0.001
Fetal total leptin [ng/ml]	2.85 (1.90–3.081)	2.89 (2.14–4.17)	0.377
Placental total leptin [pg/100 µg total protein]	130.14 (91.59–246.58)	83.84 (56.81–140.75)	0.033
Placental leptin receptor [pg/100 µg total protein]	39.70 (22.04–102.42)	36.13 (23.96–81.82)	0.660
C-Peptide [pmol/l]	44.48 (28.48–129.13)	20.91 (7.17–77.14)	0.049
Glucose [mmol/l]	5.88 (5.61–6.77)	5.66 (5.21–7.10)	0.489
HOMA = C Peptide [pmol/l] × Glucose [mmol/l]	249.31 (150.58–1010.49)	129.42 (50.72–475.43)	0.045

The data are presented as median and 25–75^th^ percentile (intequartile range).

Analyzed by Mann–Whitney *U*-test.

Differences in concentrations of fetal soluble leptin receptor, placental total leptin and of fetal C-peptide and C-peptide × glucose product (i.e., HOMA-IR equivalent) were still significant when the group of dichorionic twin pairs with two AGA children (AGA_II_, i.e., *n* = 67 twin pairs, 134 children) was included in the analysis (Table 3).

**Table 3:** Analysis of SGA twins versus AGA twin from the same pregnancy (AGA_I_) and twins from pregnancy, where the weight of both twins was appropriate for gestational age (AGA_II_).

Parameter	SGA (*n* = 32)	AGA_I_ + AGA_II_ (*n* = 166; AGA_I_ = 32 + AGA_II_ = 134)	*p*-Value
Fetal soluble leptin receptor [ng/ml]	28.63 (19.64–35.82)	19.99 (15.90–24.72)	0.0001
Fetal total leptin [ng/ml]	2.85 (1.90–3.081)	2.98 (2.39–4.18)	0.125
Placental leptin [pg/100 µg of total protein]	130.14 (91.59–246.58)	102.41 (59.94–178.58)	0.047
Placental leptin receptor [pg/100 µg of total protein]	39.70 (22.04–102.42)	47.44 (28.61–81.30)	0.776
C-peptide [pmol/l]	44.48 (28.48–129.13)	34.62 (11.16–78.77)	0.055
Glucose [mmol/l]	5.88 (5.61–6.77)	5.77 (5.22–6.58)	0.248
HOMA = C peptide [pmol/l] × glucose [mmol/l]	249.31 (150.58–1010.49)	189.97 (63.82–444.23)	0.031

The data are presented as median and 25–75^th^ percentile intequartile range. Analyzed by Mann–Whitney *U*-test.

Furthermore, the above mentioned differences were caused entirely by the SGA group, as no differences in any of the measured parameters were observed between the larger twin from the IUGR group (AGA_I_, *n* = 32) and twins from the AGA_II_ group (*n* = 134), i.e., the group where the weight of both twins was appropriate for gestational age (data not shown).

## Discussion

This is the first study to investigate the C-peptide concentration in cord blood of dichorionic twins. Clear differences in C-peptide and in C-peptide-glucose product (i.e., HOMA-IR equivalent) were found between SGA and AGA_I_ twins, thus showing evidence of insulin resistance in SGA/IUGR twins. Though these differences in C-peptide are clear, no normative data defining fetal insulin resistance currently exists, e.g., in terms of C-peptide, insulin, or HOMA-IR “cut-off points”. While one study has assessed the C-peptide concentrations in the cord blood of twins, the study involved both monozygotic and dizygotic twins and made no comparative analysis of C-peptide concentrations between twins discordant for birth weight^25^. Furthermore, our study is also the first such comprehensive assessment of the leptin system, i.e. not only total leptin, but also soluble leptin receptor as well as placental leptin/leptin receptor content.

In an unfavorable intrauterine environment resulting from too shallow trophoblast invasion into maternal decidua, the developing fetus has to employ compensative mechanisms to allow it to survive. One potential mechanism modulates glucose intake by fetal peripheral tissue to provide an adequate concentration of blood glucose, thus allowing the redistribution of glucose to organs essential for survival (“fetal salvage” hypothesis)^26^. Therefore, the development of insulin resistance could be a protective mechanism against significant depletion of glucose leading to a fall in glucose level in fetal blood.

The phenomenon of insulin resistance has been observed in SGA children born from singleton pregnancy. Takaya et al.^23^ demonstrated that the quantitative insulin sensitivity check index (QUICKI) was significantly lower in umbilical cord blood of SGA (*n* = 9) compared to AGA fetuses (*n* = 32), while a low QUICKI index (calculated from plasma insulin and glucose concentrations) indicates more pronounced insulin resistance^27^. In addition, the ADMA marker, which is depleted in patients suffering both from cardiovascular and metabolic diseases, was inversely correlated with the weight at birth in SGA children, suggesting that insulin resistance develops in SGA children during their prenatal life^23^. These data were, however, derived from singleton pregnancies. In contrast, twin pregnancies allow the inter-individual maternal factor influencing the changes in biochemical parameters to be eliminated. Furthermore, dichorionic pregnancies seem to represent an optimal model for inter-fetal comparisons than monochorionic ones: while about 10% of monochorionic twin pregnancies develop twin-to-twin transfusion syndrome (TTTS) between 16–26 week of pregnancy, this syndrome is very uncommon in dichorionic pregnancies^28,29^. The length of time when the fetus is exposed to insufficient delivery of oxygen and nutrients may play a significant role in the development of metabolic profile^30^. Therefore, while IUGR (Intrauterine Growth Restriction) syndrome occurs as a consequence of processes that take place at the beginning of the pregnancy (shallow implantation) in dichorionic pregnancies, whereas it typically arises as a consequence of TTTS in the second trimester of pregnancy in monochorionic pregnancies. According to Barker’s hypothesis, the phenomenon of metabolic programming occurs as a response of organism to nutritional stimulus or insult experienced during crucial periods of the fetal life^30,31^. Although glucose intolerance is a consequence of stress independent of its timing; it may be suspected that the earlier this stress occurs, the sooner the phenomenon of overt glucose intolerance may appear in laboratory tests.

Interestingly, the QUICKI index also appears to be positively correlated with leptin concentration^32^. Although in the present study, leptin level did not differ significantly between SGA and AGA_I_ groups, leptin access to the peripheral cells appears to be restricted in SGA fetuses. This restriction is related to elevated concentrations of soluble leptin receptor, which were observed in the present study. This receptor binds free leptin and in turn disables effective leptin action within the membrane leptin receptor. We believe that this mechanism may protect the SGA fetus against further reduction of the white fat tissue, which was emphasized in a previous animal model^19^. Moreover, this mechanism might be responsible for a reduction of insulin sensitivity of peripheral tissues, inhibiting the depletion in blood glucose below the level dangerous for fetal survival.

Our observations are consistent with those of Tzschoppe et al.^33^, who investigated both the concentration of leptin and its soluble receptor in the venous cord blood of IUGR and AGA singleton neonates at birth. Interestingly, the concentration of leptin did not differ significantly between analyzed groups; however, the level of soluble receptor was significantly higher in the population of IUGR than AGA children (*p* = 0.01). Therefore, it may be suspected that the alterations in leptin system could be related to ineffective access to nutrients and oxygen necessary for growth and organ development during pregnancy.

Another important issue pertains to higher leptin concentrations in the placentas of IUGR fetuses, without any concomitant change in the concentrations of placental leptin receptor (i.e., in this context reflecting mostly the pool of metabolically-active membrane receptors). Placental leptin appears to exert a local autocrine immunomodulatory, anti-inflammatory, pro-proliferative and anti-apoptotic role^34–36^. It has also been shown to regulate angiogenesis and induce neovascularization^37^. Therefore, we suspect that the presence of elevated placental leptin in IUGR placentas might represent an independent adaptive mechanism on the maternal side which is directed at sustaining placental function though increased leptin signaling. Such a theory is consistent with experimental models, as it has been shown that hypoxia leads to an increase expression of the active (membrane) form of the leptin receptor (Ob-R) in placental cells^38^.

There are some limitations of our study, such as the fact that the delivery occurred at various times during the night and day, and differences existed in the length of time between delivery and food and drink intake by the mother. For obstetric reasons it was, however, not possible to ensure identical condition for each delivery. There are also several methods to assess insulin resistance^39^, apart from the C-peptide-glucose product that we have employed in our study, but they were not validated for infants, so the choice of “the best” method for the time of delivery is debatable and inconclusive.

In summary, our findings demonstrate relative insulin resistance in IUGR fetuses accompanied by adaptive changes in the leptin system in IUGR fetuses and placentas from dichorionic twin pregnancies. We speculate that an increase in fetal soluble leptin receptor, potentially leading to a decrease in leptin signaling, might have a protective role against leptin-induced hypoglycemia, while a corresponding increase in placental leptin might play a role in sustaining placental function. However, it remains to be established whether the observed increase in insulin resistance might contribute to fetal metabolic programming, reflected by a decrease in post-natal insulin sensitivity and increased risk of obesity, glucose intolerance and type 2 diabetes in adults.
