# Correction: C-Peptide and leptin system in dichorionic, small and appropriate for gestational age twins—possible link to metabolic programming?

**DOI:** 10.1038/s41387-020-00135-y

**Published:** 2020-08-24

**Authors:** Krzysztof C. Lewandowski, L. Biesiada, M. Grzesiak, A. Sakowicz

**Affiliations:** 1 Department of Endocrinology and Metabolic Diseases, Polish Mothers’ Memorial Hospital—Research Institute, Lodz, Poland; 2 Medical University of Lodz, Department of Endocrinology and Metabolic Diseases, Lodz, Poland; 3 Department of Obstetrics and Gynecology, Polish Mother’s Memorial Hospital—Research Institute, Lodz, Poland; 4 Department of Perinatology, Obstetrics and Gynecology, Polish Mother’s Memorial Hospital—Research Institute, Lodz, Poland; 5 Medical University of Lodz, Department of Medical Biotechnology, Lodz, Poland

**Keywords:** Pre-diabetes, Endocrine system and metabolic diseases

Correction to: *Nutrition & Diabetes*

10.1038/s41387-020-00131-2 published online 10 August 2020

The original version of this Article was updated shortly after publication to correct two mistakes.

Under Figure 2, “The results were recalculated accordingly: nanograms [ng] of studied protein per 100 μ” should read “The results were recalculated accordingly: picograms [pg] of studied protein per 100 μg”.

In Table 2 “Fetal total leptin [ng/ml] 2.85 (1.90–3.081)” should read “Fetal total leptin [ng/ml] 2.85 (1.90–3.81)”.

Both mistakes have now been corrected in both the PDF and HTML versions] of the Article.

